# The *Community Eye Health Journal*: twenty years on

**Published:** 2008-09

**A word from former and present Editors**

