# Supplementary material for: The Community Eye Health Journal: twenty years on
Source: Community Eye Health. 2008 Sep;21(67):51. (PMC2580068)
Supplement: Related texts

# Other: DD Murray McGavin *Editor 1988–2003*

**Published:** 2008-09

**Authors:** DD Murray McGavin

**Affiliations:** Editor 1988–2003

Many years ago, in Central Asia, the *Journal* was ‘given to me to do’ – and the first issue was published in 1988. In 2003, after reaching Issue 47, the fulfilling task of Editor was handed on to a very able and experienced colleague, Victoria Francis. In 2007, Elmien Wolvaardt Ellison became our third Editor, bringing a wealth of expertise to further enhance the original vision and purpose of the *Journal.*

Through the role of Editor, I have had the opportunity and privilege of meeting and working with many colleagues and friends, truly an international community of health professionals who have sufficient vision and selfless purpose to work together, seeking to eliminate avoidable blindness worldwide.

During the past twenty years, the *Community Eye Health Journal* has sought to provide a service to health workers – not only to specialists, but to those who are simply ‘faced with eye problems’ – ophthalmologists, doctors, ophthalmic nurses, general nurses, optometrists, refractionists, ophthalmic medical assistants, ophthalmic technicians, community health workers, and many others.

It has been the ethos and purpose of the *Journal* from the outset to bring health workers up to date with current thinking and practice – a process of continuing medical and health education. In 2004–2005, a survey found that the *Journal* was the only source of up-to-date information for 73% of our readers and 72% said that the Journal had changed or influenced their practice.

**Figure:** Two eye nurses browse a copy of the *Journal.* MADAGASCAR

**Figure:** A reader survey conducted recently showed that, for 73% of our readers, this journal is their only source of up-to-date information

Perhaps appropriately, the last twenty years have seen a growth in the development of eye care services – policies for disease control, human resource development, and infrastructure and appropriate technology provision – all central to VISION 2020: The Right to Sight. It is an encouraging thought that the *Journal* has played some part in providing knowledge and disseminating information, interpreting views and policy in health care practice to its readers and, thereby, preventing blindness and visual impairment around the world.

# Other: Victoria Francis *Editor 2003–2007*

**Published:** 2008-09

**Authors:** Victoria Francis

**Affiliations:** Editor 2003–2007

My first encounter with the *Community Eye Health Journal (CEHJ)* was during its early beginnings as the ‘Bulletin of Community Eye Health’ – a simple biannual publication that aimed to extend the arm of a burgeoning training programme at the International Centre for Eye Health. The courses addressed the public health dimensions of ophthalmology and provided training relevant to resource-poor settings. As a postgraduate student at the Institute of Education, I was fascinated by the educational approach of both the courses and the extended learning materials, including the *Bulletin.* Twenty years on, the courses in community eye health and the *CEHJ*, now based at the London School of Hygiene and Tropical Medicine, continue to contribute to the global prevention of blindness. Two things strike me as remarkable about this. First, the contiguous development of the academic programme and the *Journal*, which ensures that content remains current and is continuously fed by international staff and students who contribute in many ways to its relevance. Second, the contrast in technological possibilities between ‘then’ and ‘now’. This has presented a great challenge in terms of maximising the potential of the digital revolution for those living comfortably on the digital highway, while at the same time not forsaking print as the only way to reach those without internet, who may in fact be working even beyond the end of a dirt track road. It has always been the purpose of the *CEHJ* to serve those who have limited access to information and I feel privileged to have been part of this during my period of Editorship. I wish the *CEHJ* continued success and growth in the coming 20 years!

# Other: Elmien Wolvaardt Ellison *Editor since 2007*

**Published:** 2008-09

**Authors:** Elmien Wolvaardt Ellison

**Affiliations:** Editor since 2007

What I wanted to say about the future of the *Journal* is that we hope to remain a reliable source of information and continued professional education for eye care workers who would otherwise not have access to such resources. Some news is that we will be adapting key parts of the curriculum of the MSc in Community Eye Health, which is taught at the London School of Hygiene and Tropical Medicine, as an educational series in the *Journal* (with some questions/tasks for Continuing Professional Development). This will be in the form of a four-page spread in the centre of the *Journal*, which readers can take out and keep. It will appear in every issue from 2010 until 2015. We will also keep striving to not just provide eye care information, but to provide information in a form that readers can immediately understand and use. Readers will have noticed an increase in the number of ‘boxes’ in the *Journal*, where we summarise key points and procedures as bulleted lists. We hope that some readers will cut these out and keep them handy, so they can refer to them during the course of their work – or use them to educate colleagues and students.

**Figure:** A refractionist reading a French edition of the *Journal.* MADAGASCAR
